# A Novel Anti-Influenza Copper Oxide Containing Respiratory Face Mask

**DOI:** 10.1371/journal.pone.0011295

**Published:** 2010-06-25

**Authors:** Gadi Borkow, Steve S. Zhou, Tom Page, Jeffrey Gabbay

**Affiliations:** 1 Cupron Scientific, Modi'in, Israel; 2 Microbiotest, Microbac Laboratories, Inc. Sterling, Virginia, United States of America; Saint Louis University, United States of America

## Abstract

**Background:**

Protective respiratory face masks protect the nose and mouth of the wearer from vapor drops carrying viruses or other infectious pathogens. However, incorrect use and disposal may actually increase the risk of pathogen transmission, rather than reduce it, especially when masks are used by non-professionals such as the lay public. Copper oxide displays potent antiviral properties. A platform technology has been developed that permanently introduces copper oxide into polymeric materials, conferring them with potent biocidal properties.

**Methodology/Principal Findings:**

We demonstrate that impregnation of copper oxide into respiratory protective face masks endows them with potent biocidal properties in addition to their inherent filtration properties. Both control and copper oxide impregnated masks filtered above 99.85% of aerosolized viruses when challenged with 5.66±0.51 and 6.17±0.37 log_10_TCID_50_ of human influenza A virus (H1N1) and avian influenza virus (H9N2), respectively, under simulated breathing conditions (28.3 L/min). Importantly, no infectious human influenza A viral titers were recovered from the copper oxide containing masks within 30 minutes (≤0.88 log_10_TCID_50_), while 4.67±1.35 log_10_TCID_50_ were recovered from the control masks. Similarly, the infectious avian influenza titers recovered from the copper oxide containing masks were ≤0.97±0.01 log_10_TCID_50_ and from the control masks 5.03±0.54 log_10_TCID_50_. The copper oxide containing masks successfully passed Bacterial Filtration Efficacy, Differential Pressure, Latex Particle Challenge, and Resistance to Penetration by Synthetic Blood tests designed to test the filtration properties of face masks in accordance with the European EN 14683:2005 and NIOSH N95 standards.

**Conclusions/Significance:**

Impregnation of copper oxide into respiratory protective face masks endows them with potent anti-influenza biocidal properties without altering their physical barrier properties. The use of biocidal masks may significantly reduce the risk of hand or environmental contamination, and thereby subsequent infection, due to improper handling and disposal of the masks.

## Introduction

Since the turn of the 20^th^ century, when the presence of bacteria in droplets from the nose and mouth was discovered along with their role in disease transmission, masks have been used to protect both health care providers and patients from respiratory diseases. Surgical masks are used mainly during surgery to catch the bacteria shed in liquid droplets and aerosols from the wearer's mouth and nose, and to protect the wearer from possible blood splashes. In addition to health care facilities, simple, inexpensive masks, which are similar in appearance to surgical masks, are frequently worn in crowded areas. For example, such masks were widely used, especially in China, Hong Kong, Vietnam, and Toronto, Canada, during outbreaks of the SARS virus, during the 2007 avian bird flu pandemic in Japan and, more recently, in the United States and Mexico City during the 2009 H1N1 flu (swine flue) outbreak. The use of protective masks has been shown to reduce the spread of respiratory viruses, especially when used by individuals in enclosed spaces or in close contact with a person with influenza-like symptoms [1], [2]. The USA Centers for Disease Control and Prevention (CDC) and the USA Occupational Safety and Health Administration, among others, have recommended their use to patients and health care providers [3]–[5]. Most of these masks contain a nonwoven layer that, based on its pore size, prevents the passage of pathogens through the mask, either from the environment to the wearer or from the wearer to the environment. Protective respiratory masks differ from respirators, which are devices widely used in industry to protect the wearer from noxious gases, vapors, and aerosols or to supply oxygen or doses of medication to the wearer. It is important to be aware that not all protective masks, especially those with exhalation valves, prevent passage of pathogens from the wearer to the environment. Furthermore, not all N95 respirators, especially those with exhalation valves, prevent passage of pathogens from the wearer to the environment. This is especially critical in a health care setting where a surgical respirator (a hybrid between a surgical mask and a respirator) would be needed, requiring both NIOSH and FDA approval.

The use of protective masks by the wide public received significant impetus from the World Health Organization's (WHO) declaration regarding a global flu pandemic on the 11^th^ of June 2009. This declaration came in the wake of the geographic spread of a new human influenza A H1N1 virus strain, with genes derived from human (PB1), avian (PA and PB2), classical swine (HA, NP and NS) and Eurasian swine (NA and M) influenza viruses [6]–[8]. This strain appears to have circulated in swine for years [9].

However, the efficacy of such masks is dependent on their proper use and disposal, since incorrect use and disposal may actually increase the risk of pathogen transmission, rather than reduce it [10]. The fact that healthcare workers (including doctors and nurses) have not always complied with safe disposal and handling practices, even after prolonged instruction on these practices [11], [12], is of grave concern. For example, poor hygiene practices by healthcare professionals, such as failure to wash the hands with sufficient rigor and frequency, is one of the main sources of nosocomial infections [13], [14]. Furthermore, the risks of pathogen transmission due to improper disposal and mishandling may be even greater when these masks are used by non-professionals such as the lay public [15], as is the case in the current influenza pandemic.

Copper has potent biocidal properties [16], [17]. For example, copper inactivates bacteriophages [18], bronchitis virus [19], poliovirus [18], [20], herpes simplex virus [20], [21], human immunodeficiency virus (HIV) [22]–[25] and influenza viruses [26], [27]. Recently a durable platform technology was developed, which introduces copper oxide to textile fibers, latex and other polymer products [24], [28]. The copper oxide impregnated products possess broad-spectrum anti-microbial properties [24], [28], [29] including antiviral properties [24], [25], [30].

In the present report we demonstrate that the impregnation of copper oxide into disposable N95 respiratory masks (masks that filter 95% of 0.3 micron particles) endows them with potent anti-influenza biocidal properties without altering their physical barrier properties.

## Materials and Methods

### Masks

**Figure 1:**
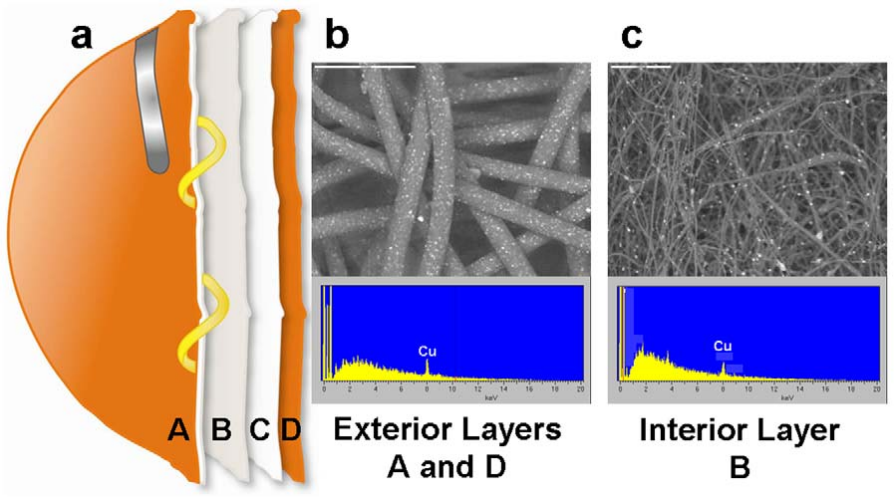
Copper oxide impregnated test mask composition. a) The test mask was composed of 2 external spunbond polypropylene layers (A and D) containing 2.2% copper oxide particles (weight/weight), one internal meltblown polypropylene layer (B) containing 2% copper oxide particles (w/w) and one polyester layer containing no copper oxide particles. b) Scanning electronic microscope picture and X-ray analysis of external layer A. c) Scanning electronic microscope picture and X-ray photoelectron spectrum analysis of internal layer B.

US National Institute for Occupational Safety and Health (NIOSH) N95 face masks containing copper oxide particles, hereafter referred to as test masks, were composed of the following 4 layers (Figure 1a): a) external layer A, made of spunbond polypropylene fabric containing 2.2% weight/weight (w/w) copper particles (Figure 1b); b) internal layer B, made of meltblown polypropylene fabric containing 2% w/w copper oxide particles (Figure 1c), which constitutes the barrier layer that provides the physical filtration properties to the mask; c) internal layer C, made of plain polyester, designed to give shape to the mask; and d) external layer D, which is identical to layer A, but is closest to the face of the wearer when the mask is used. Similar NIOSH N95 face masks, without copper oxide particles, were used as control masks and are hereafter referred to as control masks. The external layers A and D of these masks were made of spunbond polypropylene without impregnated copper oxide particles and the internal layer B was made from meltblown polypropylene without impregnated copper oxide particles. Internal layer C, made of plain polyester, was identical to that used in the test masks to give shape to the masks. The control and test masks were sterilized by gamma radiation.

### Challenge virus

Human influenza A virus (A/Puerto Rico/8/34 (H1N1)) and avian influenza virus (Turkey/Wis/66 (H9N2)) were purchased from Charles River Laboratories (Storrs, Connecticut, USA). The viral stocks were stored at −60°C to −90°C. Frozen viral stocks were thawed on the day of the test and diluted to a challenge concentration of ≥10^6^ infectious units/mL.

#### Challenge of mask with aerosolized virus

**Figure 2:**
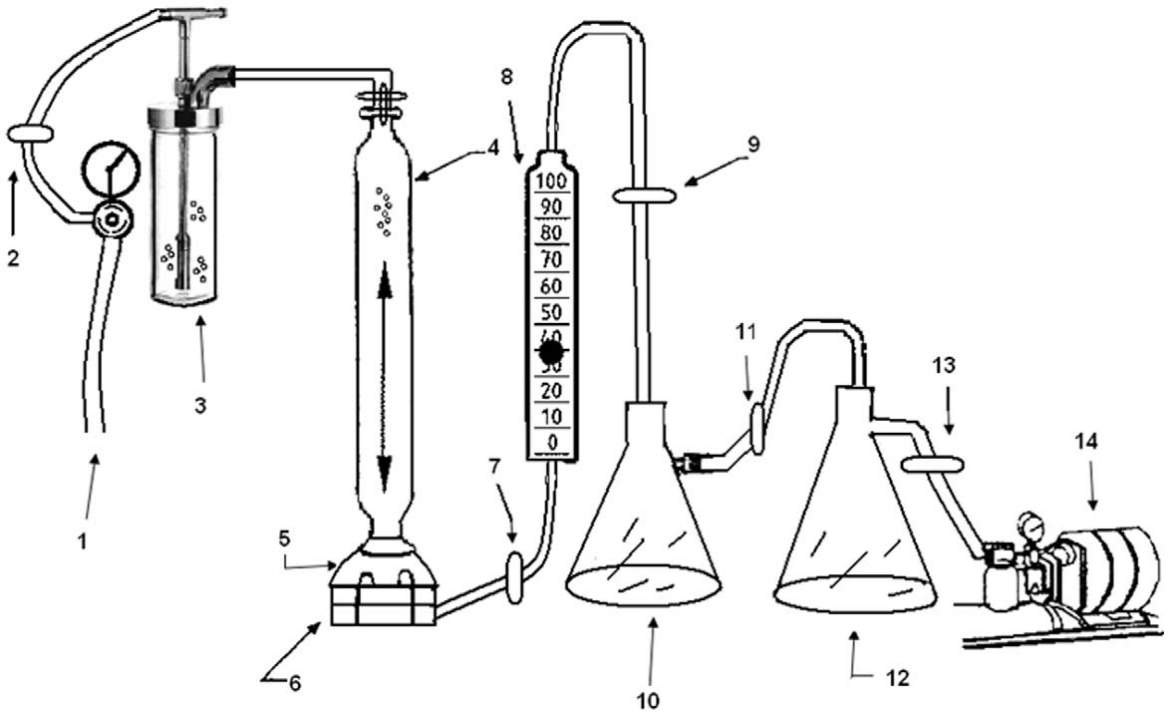
Viral aerosol challenge test apparatus scheme. Key: 1. High pressure air source; 2. Filter; 3. Nebulizer; 4. Aerosol chamber; 5. Test material chamber; 6. Anderson impactor; 7. Filter; 8. Calibrated flow meter; 9. Filter; 10. 4L vacuum flask; 11. Filter; 12. 4L vacuum flask; 13. Filter; 14. Vacuum pump.

Three control and 3 test masks were challenged with aerosolized human influenza A virus (H1N1) or aerosolized avian influenza virus (H9N2) based on the ASTM Method F 2101.01 “Standard Test Methods for Evaluating the Bacterial Filtration Efficiency of Medical Face Mask Materials, Using a Biological Aerosol of Staphylococcus aureus,” after customizing the method for virus testing. Briefly, test masks preconditioned to a temperature of 25°C and a relative humidity of ≥85% for 4 hours were hermetically clamped between a single stage Anderson impactor (Thermo Electron Corporation, Franklin, Massachusetts, USA) and an aerosol chamber under sterile conditions (Figure 2). Approximately 25 mL of human influenza A virus and avian influenza virus, respectively, were aerosolized (mean particle size: 3.0 µm±0.3 µm per manufacturer) by using a 6-jet nebulizer (BGI Incorporated, Waltham, MA USA). They were introduced into the aerosol chamber by using an upstream air compressor for 1 minute and a downstream vacuum pump attached to the impactor creating 28.3 L/min air flow through the masks. After the 1-minute challenge, the air compressor was terminated and the residual aerosol droplets were drawn through the masks for an additional 2 minutes. The aerosol droplets which penetrated the tested masks were collected from the upper surface of the stage using a flush media (Earle's Balanced Salt Solution (EBSS) +5% Newborn calf serum) and a collecting petri dish placed underneath the stage which contained semi-solid collection media (Sterile deionized water +5% gelatin +2% Bovine Serum Albumin). The semi-solid collection media were combined with the flush media and liquefied at 36±2°C for ∼15 minutes and the viral titers were determined by titration assay as detailed below. After 30 minutes the masks were aseptically removed from the impactor and transferred aseptically to a sterile stomacher bag containing 20 mL extraction medium (EBSS +5% Newborn calf serum). After stomaching, the extraction medium was transferred to appropriate sterile tubes and the infectious viral titers were determined as detailed below.

All media and equipment, including the impactor, nebulizer, scissors and forceps, were steam sterilized. All tests were conducted under a biological safety cabinet, disinfected with 70% ethanol followed by UV radiation prior to the introduction of the aerosol challenge apparatus and prior to the commencement of the experiments.

#### Determination of infectious titers

The infectious virions of each aerosol challenge as well as the retrieved virions from the control and test masks were first determined by performing ten-fold serial dilutions of each viral sample. Selected dilutions were then inoculated intra-allantoically in embryonated chicken eggs at 0.2 ml per egg and incubated for 2–4 days at 36±2°C. Four replicate embryonated eggs were inoculated per dilution tested for the titration samples. Forty replicate embryonated eggs were inoculated at the lowest dilution for the large volume samples (see below).

Following completion of the incubation period, the eggs were candled to determine viability of the embryo and then placed at 2–8°C for a minimum of 8 hours. Afterwards, the allantoic fluid was harvested and reserved at −10°C for further evaluation. These reserved allantoic samples were then assayed for the presence of virus using standard hemagglutination assay using chicken red blood cells.

The 50% Embryo Infectious Dose per ml (EID_50_/ml), equivalent to 50% Tissue Culture Infectious Dose per ml (TCID_50_/ml) in the context of this study, of the virus was determined based on the Spearman-Karber method [31].

Large volume samplings (International Conference on Harmonization (ICH), 1997; Darling, 2002) were used to increase the lower limit of detection of the infectious titers. The increase in sensitivity can be explained as follows: when samples contain a very low virus concentration, there is a discrete probability that since only a fraction of the samples is tested for virus infectivity, that fraction may test negative due to the random distribution of viruses throughout the total sample. The probability, p, that the sample analyzed did not contain infectious viruses is expressed by: p = [(V−v)/V]^y^, where V is the total volume of the container, v is the volume of the fraction being tested, and y is the absolute number of infectious viruses randomly distributed in the sample. If V is sufficiently large relative to v, the Poisson distribution can approximate p:
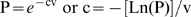
Where c is the concentration of infectious virus and v is the total sample volume. The amount of viruses which would have to be present in the total sample in order to achieve a positive result with 95% confidence (p = 0.05) is calculated as
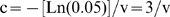
If all n replicate eggs were negative, the virus titer after the process was considered to be less than or equal to this value. The total volume of samples assayed was v = v′nd, where v′ is the test volume in a replicate, n is the number of replicates per sample, and d is the sample dilution.

A one way ANOVA and Turkey Test were used to compare between the treatments using SigmaStat 2.0 (Jandel Scientific, Richmond, CA, USA).

### Physical Barrier and Material Properties Tests

The following standard tests designed to test the filtration properties of face masks were performed by Nelson Laboratories, Inc, Salt Lake City Utah: Bacterial Filtration Efficacy (BFE) Test, Differential Pressure (ΔP) Test, Latex Particle Challenge Test and Resistance to Penetration by Synthetic Blood Test. All tests were performed using GLP procedures and in accordance with ASTM F2100-07 (Standard Specification for Performance of Materials Used in Medical Face Masks. ASTM International, West Conshohocken, PA), ASTM F2101-07 (Test Method for Evaluating the Bacterial Filtration Efficiency of Medical Face Masks Materials, Using a Biological Aerosol of *Staphylococcus aureus*. ASTM International, West Conshohocken, PA), ASTM F2299-03 (Standard Test Method for Determining the Initial Efficiency of Materials Used in Medical Face Masks to Penetration by Particulates Using Latex Spheres. ASTM International, West Conshohocken, PA), ASTM F1862-07 (Standard Test Method for Resistance of Medical Face Masks to Penetration by Synthetic Blood, ASTM International, West Conshohocken, PA) and the European EN 14683:2005, CEN/TC 205 standard (Surgical Masks – Requirements and Test Methods. European Committee for Standardization, Brussels, Belgium).

#### Bacterial Filtration Efficacy (BFE) Test

Filtration efficiency of bacterial particles is determined by comparing the challenge collected downstream of the mask sample with the known challenge delivered upstream of the mask sample. Testing was conducted both as directed in Annex B of the EN 14683:2005 standard and in compliance with ASTM F2101. Briefly, a culture of *Staphylococcus aureus ATCC #6538* (designation FDA 209 strain) was diluted in 1.5% peptone water to a concentration to yield challenge levels of 2200±500 colony forming units (CFU) per test sample. The bacterial culture suspension was pumped through a ‘Chicago’ nebulizer at a controlled flow rate and fixed air pressure (28.3 L/min; 1 cubic foot/min). The constant challenge delivery at a fixed air pressure formed aerosol droplets with a mean particle size (MPS) of 3.0 µm. The aerosol droplets were generated in a glass aerosol chamber and drawn through a six-stage, viable particle, Anderson sampler (Andersen 2000 Inc., Atlanta, GA) for collection. According to the EN 14683:2005 standard, samples were conditioned at 20±2°C and a relative humidity of 65±2% for 4 hours prior to testing. Separate samples were conditioned for 4 hours at 21±5°C and a relative humidity of 85±5% prior to testing, according to ASTM F2101. Test samples, positive controls and reference material received a one minute challenge followed by a one minute vacuum cycle. The samples were tested at normal room temperature. The outside surface of each mask sample faced the challenge aerosol. The area of each sample tested was ∼3.0 inches (75 mm) in diameter. The delivery rate of the challenge produced a consistent challenge level of 2200±500 CFU on the test control plates. A test control (no filter medium in the air stream) and reference material were included at the beginning and after the last test sample. A negative control run (without addition of bacterial challenge) was also performed. The Andersen sampler, a sieve sampler, impinged the aerosol droplets onto six soybean casein digest agar (SCDA) plates based on the size of each droplet. The agar plates were incubated at 37±2°C for 48±4 hours and the colonies formed by each bacteria laden aerosol droplet were counted and converted to probable hit values using the hole conversion chart provided by Andersen. These converted counts were used to determine the average challenge level delivered to the test samples. The distribution ratio colonies for each of the six agar plates were used to calculate the MPS of the challenge aerosol. The filtration efficacies were calculated as a percent difference between the test sample runs and the control average using the following equation: % BFE = (C−T)/C×100, where C = average of control values, and T = count of total for test material.

#### Differential Pressure (ΔP) Test

This test measures the difference in pressure through a test mask by comparing the air pressure downstream of the test mask with a known pressure upstream of the test mask. Testing was conducted as directed in Annex C of EN 14683:2005. Briefly, the ΔP test measured the differential air pressure on either side of the test sample using a manometer differential upstream and downstream of the test material, at a constant flow rate. Test samples were conditioned at 20±2°C and a relative humidity of 65±2% for 4 hours prior to testing. Separate samples were conditioned for 4 hours at 21±5°C and a relative humidity of 85±5% prior to testing. Testing was conducted at a flow rate of 8 liters per minute (Lpm)(volumetric). This value represents a corrected flow rate, which compensates for temperature and altitude differences. At least one reference material was included with each set of test samples. The ΔP was calculated using the following equation: ΔP = M/test area, where M = average mm water of test replicates. The sampler holder used in the ΔP test has a test area of 4.9 cm^2^. The ΔP value is expressed in mm of water/cm^2^ of test area when testing according to ASTM and as Pa/cm^2^ when testing according to CEN.

#### Latex Particle Challenge Test

This test is designed to evaluate pass through of very small aerosol particles (sizes between 0.1 and 5.0 microns) through the masks. The test was conducted according to ASTM F2299 in an ISO Class 5 (class 100) HEPA filtered hood. Monodispersed polystyrene (latex) microspheres of a particle size of 0.097±0.003 µm were obtained from Duke Scientific, Palo Alto, CA. These particles were nebulized, dried, and passed through the test masks. The particles passing through the test masks were enumerated using a laser particle counter. Three one-minute counts were determined for each mask sample and the results averaged. Three one-minute control counts were performed, without a test sample in the system, before and after each test sample run. More specifically, an aliquot of the latex spheres was transferred to particle free USP water and then atomized using a Particle Measuring System (PMS) Model PG-100 generator. The latex aerosol was mixed with additional filtered, dried air and passed through the test system. The flow rate through the test system was maintained at 1 cubic foot per meter (CFM)±5%. The particles delivered were collected and enumerated using a PS lased based particle counter. Extraneous particulate “background noise” through the sample holder produced an average of <1 particle at 1 CFM with the nebulizer output clamped off. A mask sample was placed into the sample holder, the system was allowed to stabilize, and then triplicate one-minute counts were recorded. Control count averages were maintained at a level of 10,000–15,000 particles per cubic foot. The percent filtration efficacy for the sample was determined using the following equation: %FE = (Average C−Average T)/Average C, where C = particle counts with no test sample in system, and T = particle counts with test sample in system.

#### Resistance to Penetration by Synthetic Blood

This procedure simulates an arterial spray and then evaluates the capacity of the material to protect the user from possible exposure to blood and other body fluids. The penetration of synthetic blood through 32 mask replicates was determined visually in compliance with ASTM F 1862. The 32 mask samples were conditioned for 4 hours at 21±5°C and a relative humidity of 85±5%. A clean canula was fixed onto the front of a valve and a reservoir was filled with synthetic blood. Each sample was tested within one minute of removing it from conditioning. Each face mask was mounted on the specimen holding fixture and positioned 305 mm (12 in.) from the canula. The mask was then subjected to the 2 ml volume spray at a pressure of 160 mm Hg, which moved from the canula in a horizontal path perpendicular to the face mask. The lab conditions during testing were a temperature of 23°C and a relative humidity of 28%. At the conclusion of the test, the backside of the mask was observed for synthetic blood penetration. The Acceptable Quality Level of this test at 120 mm Hg is 4.0%, i.e., at least 31 of the 32 masks tested needed to show no passage of synthetic blood through them.

## Results

### Controls

#### Toxicity/Negative Control

Aerosolized media containing no viruses were tested using the exact same test conditions described above in the Materials and Methods section in the absence or presence of the control and test masks secured between the sampler and aerosol chamber. Post aerosol challenge, the media were collected and diluted as described above and selected dilutions were inoculated into host egg embryos and incubated in the same manner as the rest of the test and control samples. As expected, no infectious virus was detected in the presence or absence of the control and test masks.

#### Virus Recovery Control

Human influenza A virus and avian influenza virus aliquots, containing 6.95±0.25 and 7.96±0.25 log_10_TCID_50_ units per ml (titer ± confidence limit [CL]), respectively, were used per this and subsequent tests. The average volume of the aerosolized virus delivered per run was ∼0.3 ml. The infectious titers that could be retrieved in the absence of any mask or barrier between the sampler and aerosol chamber, determined as described in the Materials and Methods section, were 5.66±0.51 and 6.17±0.37 log_10_TCID_50_ units for human influenza A and avian influenza virus, respectively. Thus all viral reduction calculations were based on these viral recovery control titers.

#### Host viability/media sterility control

Eight eggs were inoculated with an appropriate medium during the incubation phase of the study. This control demonstrated that the eggs remained viable throughout the course of the assay period. In addition, it confirmed the sterility of the media employed throughout the assay period.

#### Neutralizer effectiveness control

A test mask was challenged with mock inoculum (Earle's Balanced Salt Solution) and then put in sterile stomacher bags containing 20 mL extraction medium. After stomaching, the extraction medium was serially diluted ten-fold. Then a low level of virus (approximately 1,000 log_10_TCID_50_ units) was added to 4.5 mL of each dilution of the extraction medium (2.3 log_10_TCID_50_ units/mL). A 0.2-mL aliquot was inoculated into the host eggs as detailed in section 2.4. Infectious virus was observed in all dilutions of the extracted sample, indicating that no copper or other molecules eluted from the test masks that significantly affected subsequent influenza infection and replication in the host eggs.

### Viral Filtration by the Mask

The number of infectious human influenza A virus and avian influenza virus titers that passed through the masks (referred to as “pass through”) are shown in Table 1. Both test and control masks reduced the infectious titers that pass through the masks by ∼3 logs (2.91±1.19 and 3.55±1.14, respectively) for human influenza A virus and by ∼4 logs (4.35±0.95 and 4.12±0.64, respectively) for the avian influenza virus. No statistical significant differences between the filtration efficiencies of the test and control masks of human influenza A virus and avian influenza virus were found.

**Table 1:** Pass Through^a^ Infectious Titers.

Sample	Virus	Initial Load (log_10_TCID_50_)	Output Load (log_10_TCID_50_)	Log_10_ Reduction
Test Mask 1	H1N1	5.66±0.51	2.42±0.59	3.24±0.78
Test Mask 2	H1N1	5.66±0.51	3.40±0.28	2.26±0.58
Test Mask 3	H1N1	5.66±0.51	2.92±0.45	2.74±1.19
				Average 2.91±1.19
Control Mask 1	H1N1	5.66±0.51	2.67±0.25	2.99±0.57
Control Mask 2	H1N1	5.66±0.51	2.21±0.60	3.45±0.78
Control Mask 3	H1N1	5.66±0.51	1.83±0.31	3.83±0.60
				Average 3.55±1.14
Test Mask 1	H9N2	6.17±0.37	2.20±0.25	3.97±0.45
Test Mask 2	H9N2	6.17±0.37	2.67±0.43	3.50±0.57
Test Mask 3	H9N2	6.17±0.37	1.44±0.49	4.73±0.61
				Average 4.35±0.95
Control Mask 1	H9N2	6.17±0.37	4.90±0.00	1.27±0.37
Control Mask 2	H9N2	6.17±0.37	1.59±0.00	4.58±0.37
Control Mask 3	H9N2	6.17±0.37	2.91±0.00	3.26±0.37
				Average 4.12±0.64

a Virus that passed through the masks were recovered from both the collection petri dish and the upper surface of the stage. Here the combined viral loads, calculated by combining the viral loads from both fractions, are presented. In the cases where no virus was detected, the theoretical maximum possible load was included in the combined load as a worst-case scenario.

### Deactivation of virus remaining in the copper oxide impregnated masks

The number of infectious human influenza A virus and avian influenza virus titers recovered from the control and test masks 30 minutes after their challenge with the virus (referred to as “mask retrieved”) are shown in Table 2. In contrast to the filtration efficiencies of the test and control masks, there was a statistically significant higher direct contact inactivation of both the human influenza A virus and avian influenza virus by the test masks than by the control masks. The infectious human influenza A and avian influenza virus titers in the test masks were reduced by ≥4.78±0.88 log and 5.20±0.84 log, respectively. In contrast, the human influenza A and avian influenza virus infectious titers were reduced via direct contact by the control masks by 1.90±1.03 log and 1.34±0.84 log, respectively. The differences in the retrieved infectious titers between the test and control masks were ≥2.88 log for human influenza A (p<0.01 by ANOVA and p<0.05 by Turkey Test) and 3.13 log for the avian influenza virus (p<0.05 by both ANOVA and Turkey Test).

**Table 2:** Mask Retrieved^a^ Infectious Titers.

Sample	Virus	Initial Load (log_10_TCID_50_)	Output Load (log_10_TCID_50_)	Log_10_ Reduction
Test Mask 1	H1N1	5.659±0.51	≤0.87	≥4.78±0.51
Test Mask 2	H1N1	5.659±0.51	≤0.90	≥4.757±0.51
Test Mask 3	H1N1	5.659±0.51	≤0.88	≥4.77±0.51
				Average ≥4.77±0.88
Control Mask 1	H1N1	5.66±0.51	4.70±0.32	0.959±0.60
Control Mask 2	H1N1	5.66±0.51	3.30±0.31	2.36±0.60
Control Mask 3	H1N1	5.66±0.51	6.00±0.28	−0.34±0.58
				Average 1.90±1.03
Test Mask 1	H9N2	6.169±0.37	0.98±0.31	5.19±0.48
Test Mask 2	H9N2	6.169±0.37	≤0.97	≥5.20±0.37
Test Mask 3	H9N2	6.169±0.37	0.97±0.44	5.20±0.57
				Average 5.20±0.84
Control Mask 1	H9N2	6.16±0.37	4.50±0.00	1.66±0.37
Control Mask 2	H9N2	6.17±0.37	5.00±0.35	1.17±0.51
Control Mask 3	H9N2	6.169±0.37	5.58±0.41	0.59±0.55
				Average 1.34±0.84

a The viral load from the large volume inoculation was used as the viral load of the mask retrieved sample, since the large volume technique provides a more sensitive determination method when virus concentration was lower than the detection limit of the titration method.

### Filtration performance of the masks

Table 3 details the results obtained with the Bacterial Filtration Efficacy (BFE), Differential Pressure (ΔP) and Latex Particle Challenge standard test methods widely used in the mask industry to determine the filtration efficacy of masks. The filtration performance of the copper oxide impregnated masks met the acceptance criteria as type IIR respiratory masks, as listed in Table 1, section 5.2.3 of the EN 14683:2005 standard, and as NIOSH N-95, as follows: %BFE = ≥98%; ΔP = <49 Pa/cm^2^ and <5.0 mm H_2_O/cm^2^. In addition, blood penetration was not observed in any of the 32 masks subjected to a 2 minute spray of synthetic blood at a pressure of 160 mm Hg. This pressure is even higher than the 120 mm Hg pressure threshold of blood splash resistance required by the EN 14683:2005 standard.

**Table 3:** Filtration performance of the masks^a^.

	EN 14683:2005		ASTM F2101		ASTM F2299		
Sample	BFE (%)	ΔP (Pa/cm^2^)	BFE (%)	ΔP (mm H_2_O/cm^2^)	Average Sample Counts	Average Control Counts	Filtration Efficiency (%)
1	>99.9	41.5	98.2	4.6	1310	10438	87
2	>99.9^b^	39.5	98.6	4.3	422	10434	96
3	>99.9^b^	41.1	98.5	4.3	565	11636	95.1
4	99.2	38.1	98.6	4.3	681	12426	94.5
5	>99.9^b^	39.5	98.7	4.2	544	11153	95.1
**Mean**	99.7	39.94	98.52	4.26	704	11217	93.54
**±SD**	0.3	1.37	0.19	0.055	350	846	3.7

a Each test was done using 5 replicate masks. The result for each of the replicate mask is shown.

b There were no detected colonies on any of the Andersen sampler plates for this sample.

## Discussion

Based on a recently developed platform technology [24], [28], copper oxide particles were incorporated into 3 of 4 non-woven layers that comprise N-95 respiratory masks or Type IIR FFP1-level medical/patient respiratory masks. As demonstrated here in tests designed to simulate consumer use, the inclusion of the copper oxide particles in N-95 respiratory masks did not alter their physical filtration properties (Tables 1 and 3), but did endow them with the capacity to readily kill the virions that remain in the mask (Table 2). This is of major significance as the high viral titers that remain infectious in regular masks, as demonstrated in the control masks used in this study, can be a source of viral transmission both to the mask wearers and to others, as recently pointed out by the WHO [10].

Development of a biocidal mask (and in general, all protective personal equipment (PPE)) capable of rendering the pathogens that come into contact with them non-infectious, may significantly reduce pathogen transmission and contamination of the wearers themselves and of the environment. This may happen when healthcare workers touch their mask and then fail to wash their hands properly or at all, or when they dispose of the mask without proper safe disposal precautions. In addition to contamination from poor hand-washing practices, there is evidence of other problems such as pathogens shedding from surgical respirators onto patients in the operating theater, increasing the risk of nosocomial infections [32]–[36]. Additionally, the significantly reduced viral load remaining on the mask should protect the wearers from inhaling the virus during prolonged mask wearing.

The mechanisms of virus kill are achieved via the interaction of copper ions with the virions that are entrapped in the mask or that come into contact with the surface of the copper oxide impregnated outer surfaces of the masks. The exact copper viral kill mechanisms need to be deciphered. The capacity of copper ions to render influenza virions, including H1N1 and H9N2 viruses, non-infective has already been demonstrated [26], [27], [30]. Interestingly, it was found that the infectivity of H9N2 virus was reduced in a dose dependent manner at lower concentrations in which neither neuraminidase (NA) nor hemagglutination inhibition occurred [26]. Electron microscopic analysis revealed morphological abnormalities of the copper-treated H9N2 virus, but the exact kill mechanism was not elucidated [26].

Importantly, in addition to the antiviral properties of the copper oxide containing masks, the layers containing the copper oxide also have potent antimicrobial properties (data not shown), in accordance with the already reported broad-spectrum antimicrobial properties of fibers and fabrics containing copper oxide [24], [28], [29]. A lesser infectious bacterial load in an antimicrobial surgical respirator would at least reduce the risk of one potential source of nosocomial infections.

Could the addition of copper oxide into the masks result in an unsafe product for use? Several tests carried out in independent laboratories using good laboratory practices, which are not detailed in this report, have clearly shown that such is not the case. The amount of copper that eluted to the air from the test mask during 5 hours under simulated breathing conditions was 0.467±0.47 pg, a level that is far below (>10^5^ folds) the respiratory copper permissible exposure limit (PEL) set by the USA Occupational Safety and Health Administration (“OSHA”). The lowest observed-adverse-effect levels (“LOAELs”) for chronic copper inhalation exposure was determined to be 0.64 mg/m^3^ [37]. Again, the copper levels eluted during the simulated breathing test from the copper containing masks (0.09 pg/m^3^) are a tiny fraction (>10^6^ folds) of this copper LOAEL.

Even when simulating a worst case scenario, in which the masks would be soaked in saliva, and all the saliva would be ingested, the amount of copper eluted from the mask into the saliva was ∼7.24 µg/hr (average of three replicates minus the background), which is significantly lower than 20.8 µg/hr, the minimal risk level (MRL) for oral exposure for a person weighing 50 kg.

Importantly, the outer layers of the masks, which contain ∼2.2% copper oxide particles, did not cause any skin sensitization or skin irritation as determined in animal studies (data not shown). Also similar fabrics containing 6 times higher amounts of copper oxide did not cause any skin irritation [28]. These findings are in accordance with the very low risk of adverse skin reactions associated with copper [38] and with the lack of any adverse toxic irritations on the facial skin with ointments containing up to 20% copper [39]. In addition, the copper oxide containing masks passed flammability tests in accordance with US FDA (21 CFR Part 58) regulations, as determined in an independent FDA approved lab (Nelson Labs) using GLP.

In summary, we demonstrate that copper oxide impregnated masks safely reduce the risk of influenza virus environmental contamination without altering the filtration capacities of the masks. Due to the potent antiviral and antibacterial properties of copper oxide, we believe that these masks also confer protection from additional pathogens, and, as such, are an important additional armament in the combat against the spread of and infection by dangerous pathogens. It should be pointed out that the production of the mask layers with copper oxide and the manufacture of the mask using these materials do not add any significant costs to the price of the masks. It is suggested that copper oxide should be also included in other personal protective equipment to further confer protection to the wearer and to the environment.
